# Three pathways to better recognize the expertise of Global South researchers

**DOI:** 10.1038/s44185-023-00021-7

**Published:** 2023-08-21

**Authors:** Gabriel Nakamura, Bruno Eleres Soares, Valério D. Pillar, José Alexandre Felizola Diniz-Filho, Leandro Duarte

**Affiliations:** 1 National Institute of Science and Technology—Ecology, Evolution and Conservation Biology, Universidade Federal de Goiás, Goiânia, Brazil; 2 University of Toronto-Scarborough, Toronto, ON Canada; 3 Ecology Department, Universidade Federal do Rio Grande do Sul, Porto Alegre, Brazil; 4 Ecology and Evolution Department, Universidade Federal de Goiás, Goiânia, Brazil

**Keywords:** Scientific community, Institutions, Intellectual-property rights

## Abstract

It is widely perceived how research institutes have been adopting the discourse of champions of diversity, inclusion, and equity (DEI) in recent years. Despite progress in diversity and inclusion in the academic environment, we highlight here that nothing or, at very best, little work has been done to overcome the scientific labor division in academic research that promotes neocolonial practices in academic recognition and jeopardizes equity. In this piece, we bring secondary data that reinforce biased patterns in academic recognition between Global North and South (geographical markers and citation bias), and propose three actions that should be adopted by researchers, research institutes, journals, and scientific societies from the Global North that allows for a fairer recognition of the academic expertise produced by the Global South.

Main text

In the TV show “Better Call Saul”, the main character discovers a massive fraud case. He presents this case to a big law firm to get some help to put the case together. In response, the head of the law firm offers him a high payment but refuses to include him in the investigation. Saul refused the payment because recognizing his intellectual expertise by including him in the investigation was the priority. In a very different environment than a TV show law firm, researchers from the Global South face a parallel experience in which the more abundant funding in the Global North is applied to make a tropical science that hardly incorporates the leadership and objectives of Global South researchers. Scientific research produced by the Global South is often seen as peripherical, and Southern researchers struggle to find their expertise recognized by the Global North. While the Global North is perceived as pushing the boundaries of scientific knowledge through general theories, the Global South is often perceived as fulfilling the role of empirically testing theories, providing data, or offering fieldwork expertise^1–3^. In the worst-case scenario, empirical data obtained in Global South countries are pivotal for developing general theories led by Global North researchers, with no researcher accountability from where the data was extracted^3^. This action erases even more important contributions to the field of ecology and evolution from Global South Researchers. This global division of labor is evident when we look at geographical markers in the titles of studies for different regions of the world (Fig. 1a)^3^ (any spatial delimitation, but here represented only by country names). The zoogeographical division of the world^4^ also carried imprints of biases, with the Neotropics and Afrotropics showing disproportional mentions in the titles of the studies analyzed here (with 51 and 2 mentions, respectively), evidencing a global demarcation also reflected in natural boundaries.

**Fig. 1:**
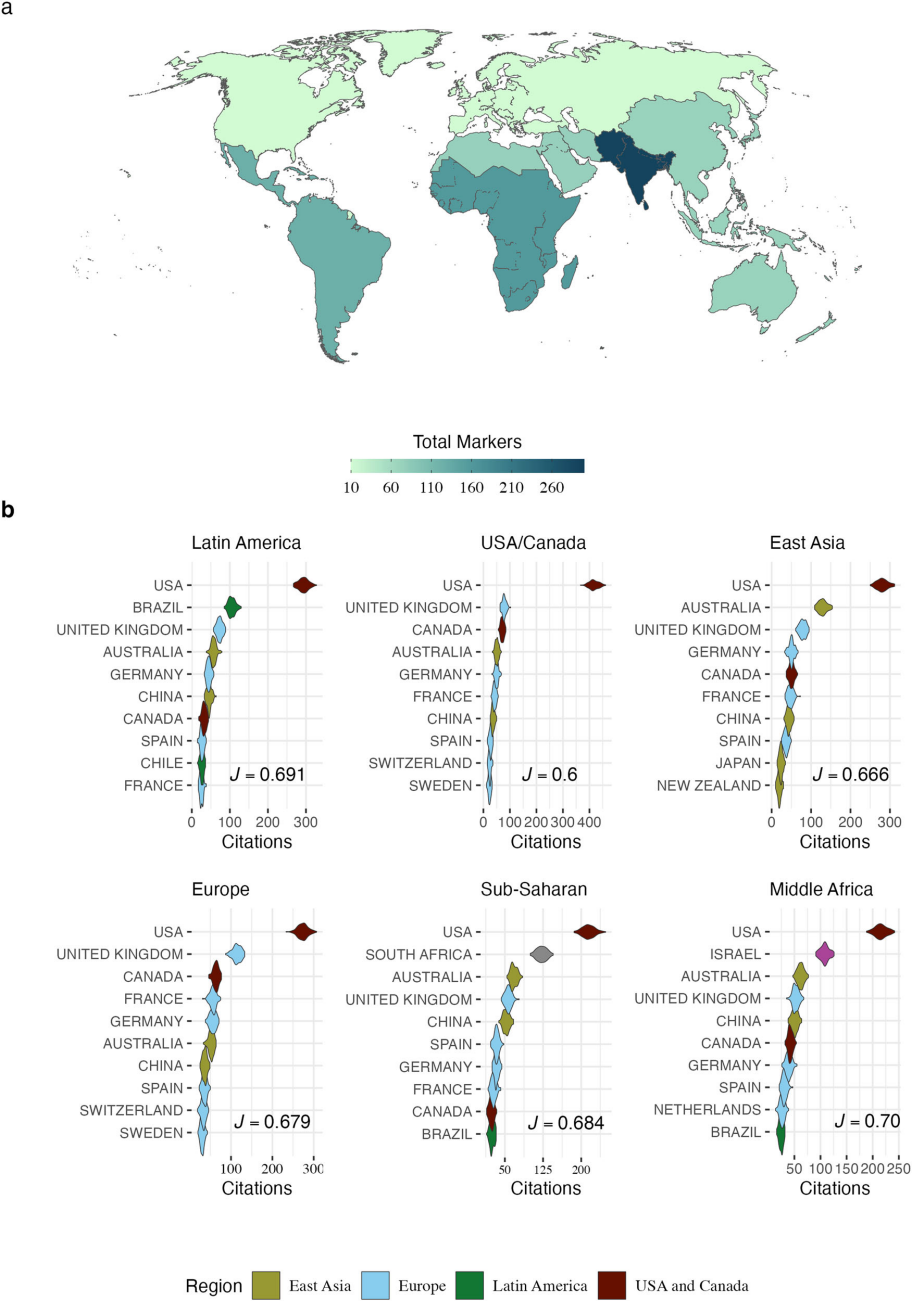
Different aspects of academic recognition. At the top, a map (**a**) showing the number of times country names appeared in the article titles produced by each region. For all figures, we used data from the top 1000 articles in high-ranked Ecology and Evolution journals for each world region (see Data Availability at the end for a complete list of journals). World region was defined accordingly to the World Bank classification of the countries. Violin charts (**b**) represent the rarefied values (based on 1000 sampled articles) of the number of times articles published in each region (Latin America, USA and Canada, East Asia, Europe, Sub-Saharan, and Middle Africa) were cited in articles published by authors affiliated with institutions in different countries (from 1945 to 2023). On the bottom right of each violin chart is the Pielou evenness index; the lower the value, the more biased towards a given country, citing the papers produced in a given region disproportionally.

Diversity, equity, and inclusion (DEI) have been a central part of the academic debate in the last few years, driving cultural and structural changes in research institutes and universities of the Global North. A significant focus at the Global North has been on diversifying the pool of applicants by encouraging applicants from underrepresented backgrounds to apply and promoting the debate on building inclusive teams^5,6^. While these actions are essential to advance DEI in the Global North academic ecosystem, they seldom change global resource and academic recognition disparities.

Global North researchers are often regarded as experts in their respective fields, enjoying a reputation beyond their local contexts. Conversely, Global South researchers are often perceived as being confined to their own regions, with their scientific authority seen as deriving from the knowledge and expertise originating in the Global North. The perception that expertise flows from the Global North to the Global South is maintained by deeply rooted practices in academia, creating the colonial structure of academic knowledge. Another example of academic neocolonialism is the bias in citations^7,8^ and claims of scientific discovery. Recognition of scientific achievements are usually measured through the number of citations (despite the controversies around this measure)^9^. However, it is common that papers with novel insights or findings published by researchers or institutions from the Global South are less cited in studies from research groups from the Global North, even publications presented in long-standing, high-impact journals^7^. This situation creates a vicious cycle in which northern institutions, mainly in Europe and North America, dictate knowledge, maintaining the *status quo* of academic expertise. Other examples include practices of data acquisition with no engagement of local knowledge (known as parachute science), and the underrepresentation (or complete lack of representation) of scientists from the Global South as speakers in conferences and editorial boards of long-standing journals^6^. Some mechanisms maintaining this structure include taking English as the *lingua franca* of scientific practice^10^ and even positive bias for Global North countries to publish in (their own) high-impact journals^11^.

While we acknowledge the recent progress in DEI in academia (e.g., Brazil receiving more citations in some regions than European countries in Fig. 1), little or nothing has been done to reduce the practices that promote the global academic labor division that frames Global South researchers as primarily data gatherers or case study producers. Overcoming this neocolonial structure implies recognizing the knowledge produced outside the Global North as being as reliable and scientifically sound as the one made by research institutes in the Global North. Scientific solutions require specific and contextual knowledge, especially in the face of global changes^12^. For example, management actions and policies developed to protect and maintain biological diversity and ecosystem services might not be the same in tropical and temperate regions^13^. Also, the values that different communities hold might require different responses and debates with local and global science^14^. Consequently, excluding the scientific knowledge produced in those places is rooted in academic colonialism and should be considered to develop better solutions.

Here, we argue that if the Global North is committed to changing the *status quo* of academic knowledge, researchers and research institutes must do a better job toward actions that improve the intellectual visibility of underrepresented groups by (i) recognizing practices in scientific work that promote intellectual neocolonialism and (ii) implementing actions that break down the labor division in scientific knowledge. In the following sections, we propose interventions that the Global North, from individuals to institutions, should adopt to support a contra-colonial structure knowledge production.

## Some suggestions from the Global South

It is known that the notion of privilege is usually unrecognized by those who are privileged by it^15^. Therefore, we delve into the idea that the change must come from the oppressed, and we, the Global South researchers, should be the ones driving changes in our scientific practice^16,17^. The evidence shows that the Global South is the one acting towards a more equitable science by promoting a more equitable academic recognition (expressed by the higher equitability in citation proportion in Fig. 1, Latin America, Middle Africa, and Sub-Saharan violin chart). Nevertheless, to be effective, structural changes in the global academic system must be carried out by the entire community. Here, we cite simple actions that could be taken to mitigate intellectual neocolonial practices in science and further recognize the expertise of researchers from the Global South. Despite most of our suggestions being derived from Ecology and Evolution examples and limited by the author’s backgrounds, we believe they can be applied to other scientific areas.

### Action 1: Increasing diversity in scientific groups (journals, societies, and boarding members of scientific meetings/events)

*Why does it matter?* Since board members of conferences, editorial boards, and societies are a non-random sample of ecological researchers and experts^18^, their decisions are biased at certain extensions to their personal experiences and backgrounds. Therefore, increasing the participation of historically excluded groups improves the decision-making process by amplifying and considering different points of view with diverse backgrounds and perspectives.

*What to do?* Journals and scientific societies must diversify their editorial and committee boards by including researchers from historically marginalized groups. Their participation in these spaces would help identify and tackle specific problems faced by people from different backgrounds. For example, non-native English speakers face additional barriers to publishing papers in English-only journals because of language^10^. In response, the Society for the Study of Evolution provides cost-free English language editing for non-native English-speaking authors, reducing the language barriers to scientific publication^19^.

### Action 2—Reducing costs of open-access publications

*Why does it matter?* Most publishers do not provide waivers for developing countries, making open access a privilege for Global North researchers^20^. For example, if the open access fee of a given journal is 4000 United States dollars, this would be equivalent to almost two monthly wages of an assistant professor in Brazil. Even when waivers are provided, the cost is often prohibitive, excluding most researchers from the Global South from taking part in more globalized publishing venues.

*What to do?* Provide more waivers for Global South researchers and actively pursue partnerships with Global North institutions to cover fees.

### Action 3—Referencing the Global South expertise

*Why does it matter?* Modern science requires finding solutions that are adequate for different contexts. Students and researchers are exposed mainly to the science produced by the Global North in their curricula, texbooks^21^, and articles, providing them with a limited overview of potential solutions to global problems. For students and researchers, amplifying their sources or information to the Global South increase the capacity for generalization, the understanding of contextual environmental and socioeconomic factors affecting biodiversity, and social engagement^22^. For researchers at the Global South, the increasing recognition of our work means increasing citations that might boost our careers and potential collaboration with Global North researchers^12^.

*What to do?* Researchers and professors should familiarize themselves with the literature produced by Global South researchers, especially when working with tropical ecology. Authors from the Global North must check if their references do not neglect relevant articles and examples from the Global South. Publishers and editors might demand more globalized examples or suggest literature when necessary. A starting point could be explicitly encouraging reviewers to be aware of possible citation bias.

## Towards a contra-colonial science

Research institutes in the Global South still have a long way ahead when compared with the Global North institutes regarding the number of publications (in terms of quantity), and different factors can explain this (including local conditions of research institutes in the Global South). However, in terms of quality, numerous examples of universities and research groups of excellence in the Global South are a reference in different areas of Ecology and Evolution (not to mention other areas of STEM), even struggling with reduced budgets and various forms of historical colonialism. Here we suggested three simple actions that can dramatically change the *status quo* of scientific knowledge. Recognizing intellectual colonialism practices is the first step, but not enough if scientific practitioners aim to build a truly inclusive environment and reduce inequalities. We can learn from the great Brazilian educator and philosopher Paulo Freire that *praxis*, i.e., “reflection and action upon the world in order to transform it,” is the only way toward a non-oppressive, inclusive, and diverse science. True changes in an oppressive system can only come from those who have been oppressed, but for this, the Global South needs to take a seat at the same table as the Global North already has.

## Positionality statement

We acknowledge that the views, opinions, and suggestions presented here are not exhaustive in addressing the issue of colonial practices in the science of ecology and evolution. We are not free from bias in approaching this subject, particularly evident in the lack of gender equality and the exclusive representation of Brazilian researchers in this piece. Therefore, we recognize the limitations of our opinions. However, we believe that our suggestions are grounded in a body of evidence derived from secondary data and existing literature on the topic. As authors, we come from diverse contexts, training experiences (including those trained in the Global South and based in the Global South, trained in the Global North and based in the Global South, and trained in the Global South and based in the Global North), and career stages (ranging from early career to senior researchers). Despite our varied backgrounds, we collectively agree that the science of ecology and evolution must prioritize inclusivity and provide fair recognition to scientists from the Global South through actions. It is essential to address the structural problems rooted in colonial practices to achieve a comprehensive solution.

## Data Availability

All data used to produce Fig. 1 was collected in the Web of Science Core collection between December 2022 and February 2023. All data used to make the Fig. 1 are available in the 10.5281/zenodo.8034469.

## Code availability

All codes and scripts used to make the queries in Web of Science Core collection, process and analyze the data are available in the GitHub repository (https://github.com/GabrielNakamura/Decolonizing_expertise), and also in the Zenodo repository under the 10.5281/zenodo.8183465.
