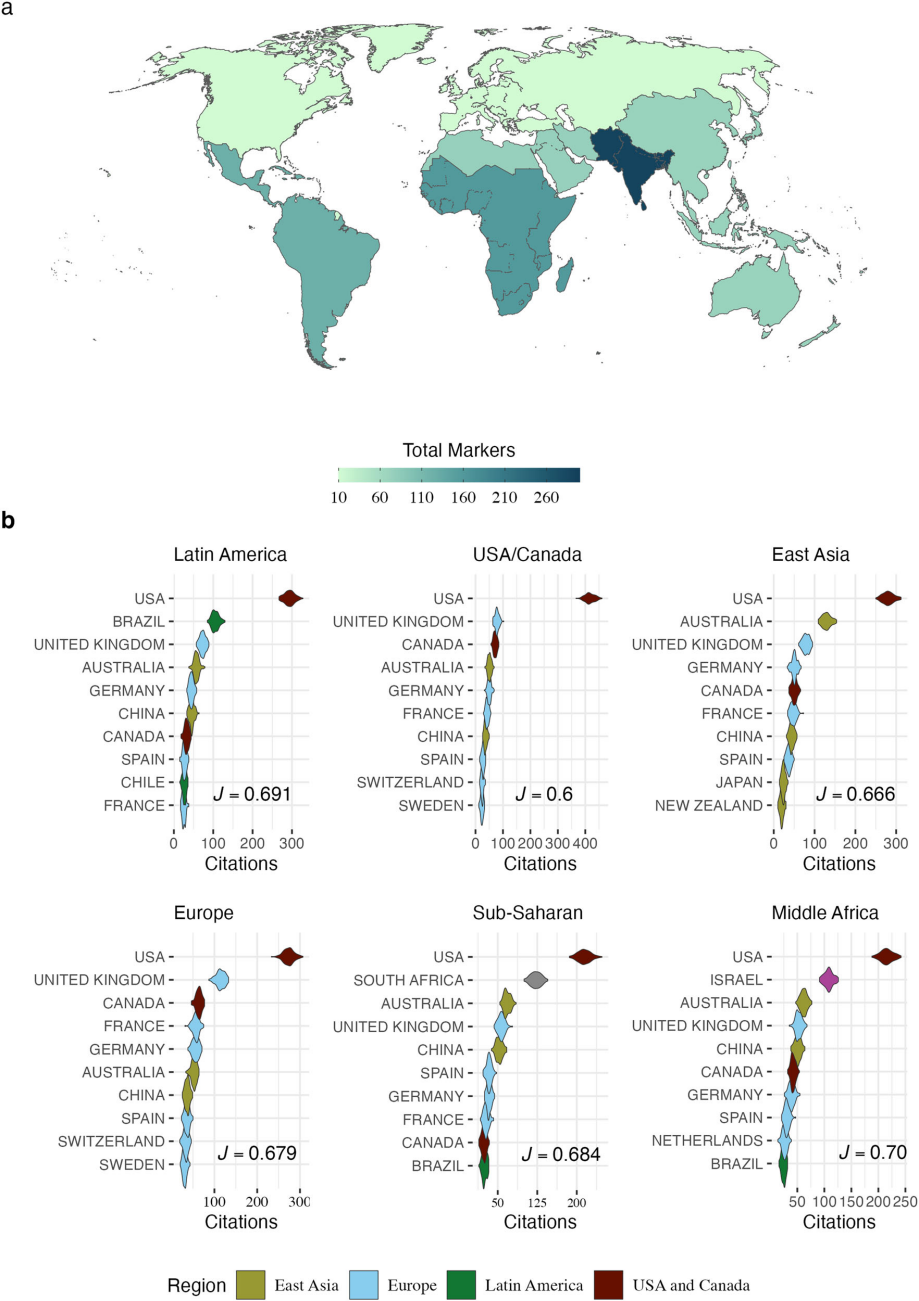# Author Correction: Three pathways to better recognize the expertise of Global South researchers

**DOI:** 10.1038/s44185-023-00025-3

**Published:** 2023-09-12

**Authors:** Gabriel Nakamura, Bruno Eleres Soares, Valério D. Pillar, José Alexandre Felizola Diniz-Filho, Leandro Duarte

**Affiliations:** 1 National Institute of Science and Technology—Ecology, Evolution and Conservation Biology, Universidade Federal de Goiás, Goiânia, Brazil; 2 University of Toronto-Scarborough, Toronto, ON Canada; 3 Ecology Department, Universidade Federal do Rio Grande do Sul, Porto Alegre, Brazil; 4 Ecology and Evolution Department, Universidade Federal de Goiás, Goiânia, Brazil

**Keywords:** Scientific community, Institutions, Intellectual-property rights

Correction to: *npj Biodiversity* 10.1038/s44185-023-00021-7, published online 21 Aug 2023

In the original version of this Comment, the authors mistakenly omitted the South Asia region in Fig. 1. This has now been corrected in the PDF and HTML versions of the Article.